# PCR Characterization of Microbiota on Contracted and Non-Contracted Breast Capsules

**DOI:** 10.1007/s00266-019-01383-9

**Published:** 2019-05-02

**Authors:** Yara Bachour, Linda Poort, Stephan P. Verweij, Gijs van Selms, Hay A. H. Winters, Marco J. P. F. Ritt, Frank B. Niessen, Andries E. Budding

**Affiliations:** 1 Department of Plastic, Reconstructive and Hand Surgery, VU University Medical Center, De Boelelaan, 1007 MB, 1117 PO Box 7057, Amsterdam, The Netherlands; 2 Department of Medical Microbiology and Infection Control, VU University Medical Center, Amsterdam, The Netherlands; 3 Department of Plastic, Reconstructive and Hand Surgery OLVG Location West, Amsterdam, The Netherlands

**Keywords:** Capsular contracture, Aetiology, Breast implants, Breast augmentation, Bacteria, Immunology

## Abstract

**Background:**

The aetiology of capsular contracture around breast implants remains unclear. The leading theory is that a subclinical infection around the implant plays a role in the development of capsular contractions. Several studies found associations between the presence of bacteria and the occurrence of capsular contraction. However, it is unclear whether detected bacteria originate from the breast capsule, breast glandular tissue or skin contamination. Moreover, this has never been investigated with molecular techniques. The aim of this study was to assess the bacterial microbiota on breast capsules, glandular tissue and skin using a highly sensitive PCR assay.

**Materials and Methods:**

Fifty breast capsules were collected during implant removal or replacement. Ten specimens of glandular breast tissue and breast skin were collected in females who were undergoing reduction mammoplasty. A sample specimen (4 mm) was sterilely obtained from all tissues. All specimens were analysed by IS-pro, a 16S–23S interspace region-based PCR assay.

**Results:**

Low numbers of *Staphylococcus spp.* (four species in four capsules) were found on breast capsules. There was no difference in bacterial presence between normal and contracted capsules. The skin of the breast-harboured *Streptococcus spp. and Staphylococcus spp*. while the glandular tissue was sterile.

**Conclusion:**

The low numbers of bacteria found on the capsules are most likely caused by contamination during capsule removal. More and larger studies are needed to investigate the bacterial presence on breast capsules using a PCR assay. This is the first study in which breast capsules have been studied using a highly sensitive PCR assay.

**Level of Evidence IV:**

This journal requires that authors assign a level of evidence to each article. For a full description of these Evidence-Based Medicine ratings, please refer to the Table of Contents or the online Instructions to Authors www.springer.com/00266.

## Background

For decades, breast implants have been used for breast augmentation or breast reconstruction after breast cancer [1]. As a normal foreign body reaction, a fibrous capsule is formed around the breast implants [2–4]. This capsule is usually thin and not visible on the outside, but in some cases, this capsule tends to harden and tighten around the implant, causing capsular contracture. This complication presents symptoms of pain, hardening, thickening and disfiguring of the breast. Capsular contracture is the most frequent complication after breast augmentation or reconstruction with breast implants [1, 5–7]. The prevalence varies between 5 and 19% [8–10] for breast augmentation and 19 and 25% [9, 11, 12] for breast reconstruction, which makes it the primary reason for reoperation after breast implant implantation [10]. The degree of capsular contracture is clinically graded by the Baker classification system ranging from Baker 1&2 (normal capsule) to Baker 3&4 (capsular contracture) [13].

To date, the aetiology of capsular contracture is unknown [14]. It is thought to be a multifactorial condition consisting of immunobiological factors such as a subclinical condition and/or altered immune response but also of several patient-, surgery, and implant-related risk factors [15]. One of the prominent immunobiological theories is that subclinical infections around the implant play a causative role in its development. Several human and animal studies have found associations between the presence of bacteria and the occurrence of capsular contraction [16–19]. Specifically, the most commonly detected bacteria on contracted capsules were *Staphylococcus spp.* This theory of a subclinical infection has also been supported by studies that show a reduction in capsular contracture after administration of antibiotics prophylactically or postoperatively [16, 20].

Although previous studies strongly suggest a causative role for bacteria in the development of capsular contraction, they failed to demonstrate a clear association between bacteria and capsular contracture due to the heterogeneity of the studies and suboptimal sterile sampling conditions. Therefore, it is currently unclear whether detected bacteria originate from the breast capsule, glandular breast tissue or skin contamination. Furthermore, all studies used culture methods to detect bacteria. Although culture is the gold standard for detecting bacteria, it is restricted to the cultivable fraction of bacteria. Currently, sensitive molecular polymerase chain reaction (PCR) methods are available that can detect a much broader range of bacteria [21–23].

The aim of the present study was therefore to assess the microbiota according to a sterile regime on normal and contracted breast capsules using a highly sensitive PCR assay (the IS-pro assay), which identifies bacteria by measuring the length of the 16S–23S region [24]. Additionally, this assay was used to assess the endogenous microbiota of the glandular tissue of the breast as well as the breast skin.

## Materials and Methods

This was a cross-sectional study. Patient characteristics were retrospectively collected. Samples were collected between 2014 and 2016 at the VU Medical Center, Jan van Goyen and the OLVG West location. The local medical ethical committee approved this study (reference number: 2014.110 and 2014.146). All participants provided written informed consent.

### Sample Collection

Normal and contracted capsules were collected to investigate the microbiota on breast capsules. We included females who underwent implant replacement or removal for any reason. The subjects were treated according to the normal surgical procedures and received cefuroxime 1000 mg preoperatively. In all patients, the Baker score, as used in clinical practice [13], was determined by two physicians who together reached an agreement together. Baker scores of 1 and 2 were considered normal capsules, while Baker 3 and 4 were considered capsular contractures. The capsules were removed by the surgeon within the first 10 min of the operation using a cauterizer under sterile operating conditions. All capsules were taken at the site of incision at the inframammary fold. Special care was taken to avoid any contact of the capsules with the breast skin. A sample specimen (4 mm) was obtained from the removed capsules using a fresh, sterile scissor and tweezer at a sterile table. Afterwards, the specimens were collected in sterile specimen containers followed by immediate snap-freezing in liquid nitrogen and stored at − 20 °C until further analysis.

Females were included in the study who underwent reduction mammoplasty and had no history of prior breast surgery or a history of breast infection to investigate the microbiota of the glandular tissue. These females were treated according to normal surgical standards and received 1000 mg cefuroxime i.v. preoperatively. Before preparing the skin with chlorhexidine, a skin area of 3 × 3 cm was sampled with a swab (Copan flocked swab 552C moistened with 200 µl reduced transport fluid) at the site of incision. The breast tissue was removed by the surgeon under sterile operating conditions. A sample specimen (4 mm) was obtained from the glandular tissue using a fresh, sterile knife and tweezer at a sterile table. Both specimens were collected in sterile specimen containers and stored within two hours at -20 °C until further analysis. All samples were collected, stored and transported by one and the same investigator according to the aforementioned protocols.

### Laboratory Testing

Bacterial DNA was extracted from glandular breast tissue and capsule biopsy specimens by a first step consisting of lysis of bacteria. Biopsies measuring 4x4 mm were cut to pulp before adding 1 ml of easyMAG (BioMérieux, Marcy l′ Etoile, France) lysis buffer. This mixture was vortexed and incubated at room temperature while shaking at 1400 revolutions per minute (RPM) for 10 min. After a centrifugation step of 2 min at 14.000 RPM, the supernatant was used for DNA extraction on the easyMAG automated DNA isolation machine (BioMérieux). DNA was eluted in 70 µl NucliSens easyMAG extraction buffer 3 as provided by the manufacturer (BioMérieux), choosing the machine’s regular program with external lysis, according to the manufacturer’s instructions. Positive controls were added for each DNA isolation run. Amplification of IS-regions was performed with the IS-pro assay (IS-diagnostics, Amsterdam, the Netherlands) according to the protocol provided by the manufacturer. IS-pro differentiates bacterial species by the length of the 16S–23S rDNA intergenic spacer (IS) region with taxonomic classification by phylum-specific fluorescently labelled PCR primers (2). The procedure consists of two separate standard PCRs: the first PCR contains two different fluorescently labelled forward primers targeting different bacterial groups and three reverse primers providing universal coverage for those groups. The first forward primer is specific for the phyla Firmicutes, Actinobacteria, Fusobacteria and Verrucomicrobia (FAFV), and the second labelled forward primer is specific for the phylum Bacteroidetes. A separate PCR with a labelled forward primer combined with seven reverse primers is specific for the phylum Proteobacteria. Amplifications were carried out on a GeneAmp PCR system 9700 (Applied Biosystems, Foster City, CA). After PCR, 5 μl of PCR product was mixed with 20 μl formamide and 0.2 μl custom size marker (IS-diagnostics). DNA fragment analysis was performed on an ABI Prism 3500 Genetic Analyser (Applied Biosystems). Data were analysed with the IS-pro proprietary software suite (IS-Diagnostics, Amsterdam, the Netherlands), and the results are presented as microbial profiles. Automated species calling of IS-pro peaks was performed with the dedicated IS-pro software suite (IS-Diagnostics) in which peaks are linked to a database containing IS profile information of > 500 microbial species. Peaks lower than 128 relative fluorescence units (RFU) were regarded as background noise and were discarded from further analysis.

### Data Processing

Data were further analysed with the Spotfire (TIBCO, Palo Alto, CA, USA) software package. Descriptive statistics are provided and presented as number (%) and mean (SD). Student’s *t* test was used for continuous data, and *p* values less than 0.05 were considered statistically significant. Statistical tests were performed using SPSS 22.0 (IBM SPSS Statistics for Windows, Version 22.0. IBM Corp., Armonk, NY, USA).

## Results

### Subjects and Implant Characteristics (Normal and Contracted Capsules)

We included 50 breasts from 26 subjects. The breast capsules originated from cisgender females, with the exception of one male-to-female transgender subject. The mean age at the time of operation was 46 (SD 12.0) years. The primary indication for breast implantation was, in most cases, cosmetic (94%) and, in a few cases, for reconstructive surgery after breast cancer (6%). The Baker score was grade 1 in 12 cases, grade 2 in 16 cases, grade 3 in ten cases and grade 4 in 12 cases (see Table 1). The implants were removed after a mean of 11 (SD 5.6) years. The implantation duration for each Baker score was as follows: Baker 1: 6.6 years (SD 5.0 years), Baker 2: 11.5 years (SD 5.5 years), Baker 3: 8.8 years (SD 4.9 years) and Baker 4: 15.3 years (SD 4.0 years). This was significantly higher for higher Baker scores: Baker 1 vs. Baker 2 (*p* = 0.033), Baker 1 vs. Baker 4 (*p* = 0.002) and Baker 3 vs. Baker 4 (*p* = 0.017).

**Table 1:** Patient characteristics

	Study 1: endogenous microbiota of the skin and the breasts’ skin *n* = 10 (%)	Study 2: normal and contracted breast capsules *n* = 50 (%)
Mean age in years (SD)	51 ± 7.4	46 ± 12.0
Implant duration in years (SD)	n/a	11 ± 5.6
(Primary) indication for operation		
Reduction	10 (100)	n/a
Augmentation	n/a	47 (94)
Reconstruction	n/a	3 (6)
Baker score		
1	n/a	12 (24)
2	n/a	16 (32)
3	n/a	10 (20)
4	n/a	12 (24)

*n* number of patients, *SD* standard deviation, *n/a* not applicable

### Subjects (Glandular Breast Tissue)

We included ten females receiving reduction mammoplasty due to breast hypertrophy. The mean age during operation was 51 (SD 7.4) years.

### Normal and Contracted Capsules

**Fig. 1:**
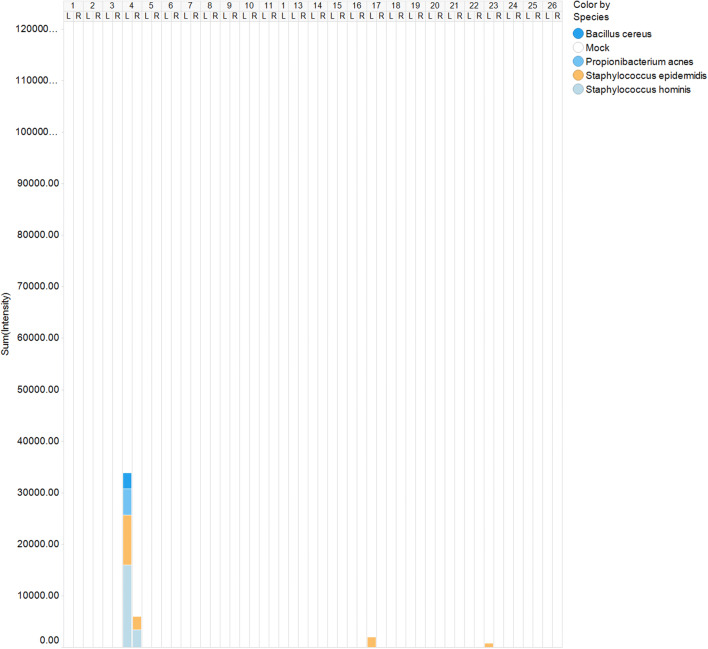
IS profiles of 50 capsules, including both normal and contracted capsules. The (cumulative) intensity is expressed in RFU, which reflects the quantity of bacteria present

Both normal and contracted capsules were almost always sterile. In both groups, very low amounts of *Staphylococcus spp.* (*S. epidermidis* (4/50) and *S. hominis* (2/50)), *Propionibacterium acnes* (1/50) and *Bacillus cereus* (1/50) were detected (Fig. 1). There was no difference in bacterial presence between normal and contracted capsules (*p* = 1.0).

### The Endogenous Microbiota of the Breast and the Breast Skin

**Fig. 2:**
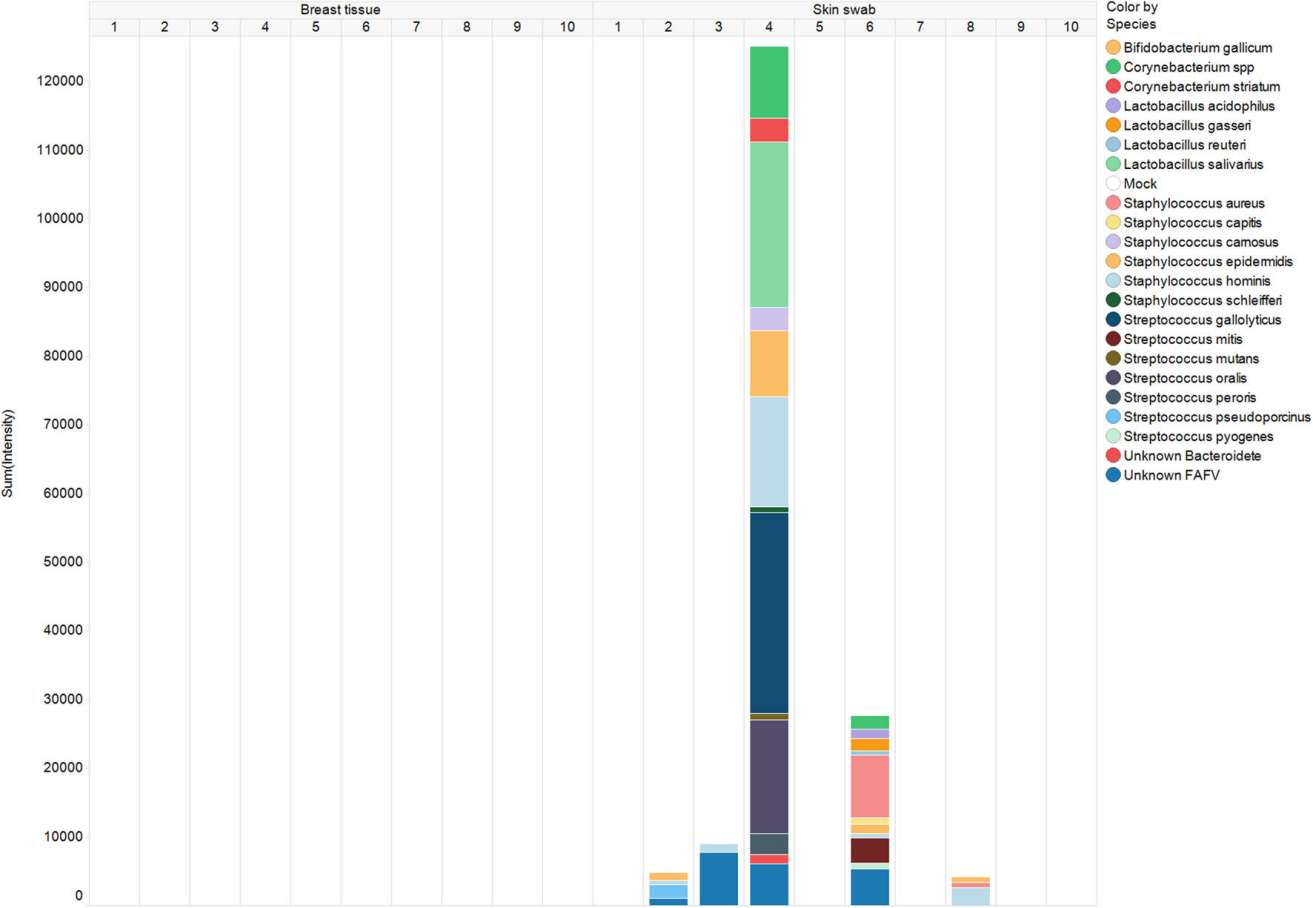
IS profiles of ten samples of breast skin. The (cumulative) intensity is expressed in RFU, which reflects the quantity of bacteria present

The skin of the breast mainly harboured *Streptococcus spp. and Staphylococcus spp.* while the glandular tissue of the breasts was sterile (Fig. 2).

## Discussion

The aim of this study was primarily to investigate the bacterial microbiota on normal and contracted breast capsules using a highly sensitive PCR assay for the detection of bacteria. Using this sensitive PCR assay, we also investigated the endogenous microbiota of the glandular tissue and skin of the breasts. The current study found that normal and contracted breast capsules as well as glandular breast tissue are generally sterile. In normal and contracted breast capsules, we found hardly any bacteria with the exception of very low numbers of common skin bacteria. Common skin bacteria were found on the breast skin, while we did not find any bacteria in glandular tissue.

In the present study, we found normal and contracted capsules to be generally sterile. Six studies have investigated the role of bacteria in the development of capsular contracture [16–18, 25–27]. One study found no bacteria [25], while the remaining studies found bacteria on normal as well as contracted capsules [16–18, 26, 27]. In all of these studies, cultured capsules resulted in higher numbers of positive cultures in contracted capsules than in normal capsules. In some cases, this difference was even significant [18, 26, 27]. The most common bacteria in these positive cultures were *Staphylococcus spp.*, mainly *S. epidermidis* (ranging from 17.8 to 84%). The hypothesis that bacteria play a role in the development of capsular contracture has also been investigated by administrating antibiotics prophylactically or postoperatively, which has yielded contradictive results [16, 20, 28–35]. The aforementioned studies suggest that bacteria might play a role in the development of capsular contracture.

Nonetheless, there are several issues concerning the studies in which capsules were cultured. First, it is difficult to draw definitive conclusions based on these studies due to the heterogeneity of included patients, heterogeneity of surgical procedures, differences in culture media and long follow-up periods. Second, most studies have used suboptimal sterile sampling conditions and do not follow or mention strict sterile sampling conditions. Therefore, the likelihood of contamination of the samples increases. Third, all of these studies used culture to show the presence of bacteria [16–18, 25–27]. A recent study showed that the IS-pro technique detected up to 47% more species in comparison with culture. The IS-pro technique has been validated to detect bacteria from the phylum Firmicutes and Actinobacteria (to which the species found in previous studies belong) up to at least ten colony-forming units (cfu) [36].

The low numbers of *S. epidermidis* that were detected on breast capsules suggest that these bacteria might originate from contamination of the capsules with skin bacteria during surgical removal. Much higher numbers of bacteria would have been expected in the case of bacterial infection of the breast capsules. Indeed, foreign object infections caused by *S. epidermidis* are characterized by biofilm formation in which bacterial loads are generally very high [37].

Moreover, *S. epidermidis* is a commensal inhabitant of the skin, so it is not unlikely that some biopsies were contaminated with *S. epidermidis* during surgical removal [38]. This would also explain the high number of bacteria found in former studies using culture techniques. Contaminated capsules could show exponential growth of skin bacteria during culturing. With our technique, positive controls were added to each DNA isolation run to assess whether the IS-pro assay recognizes microbial presence in the collected samples. Since all of the positive controls tested positive, we believe that the samples presumed to be sterile were indeed sterile and that the low numbers of bacteria present are likely to represent contamination of the samples with skin bacteria.

If bacteria in situ play a role in the aetiology of capsular contracture, it is likely that this would have been shown not only using technical evidence but also in clinical symptoms. None of our patients reported clinical symptoms of an ongoing or chronic infection, such as (intermitted) fever or fistula. These clinical symptoms have not been reported in former studies investigating the role of bacteria in the aetiology of capsular contracture [16–18, 25–27].

To investigate the origin of a potential bacterial contamination in capsular contracture, we assessed the breast skin and the endogenous microbiota of the breast. Several studies have assessed the microbiota of breast tissue [39–45]. Ransjö et al. [39], Tharnton et al. [40] and Bartsich et al. [41] investigated the endogenous microbiota of breast tissue using culture techniques. The most abundant bacteria in all three studies were *S. epidermidis/*coagulase–negative *Staphylococcus*, ranging from 15 to 90%, and *P. acnes*, ranging from 11 to 48%. We found no bacteria in the glandular tissue of the breast using our technique. However, we investigated deep glandular breast tissue. Previous studies on the microbiota of breast tissue do not precisely mention which part of the breast tissue was analysed. It is possible that deep glandular tissue is farther away from the skin and therefore harbours less bacteria than superficial tissue. Glandular tissue also contains antimicrobial peptides such as human β-defensin-1 [46, 47] and cathelicidin [48], which could explain our sterile samples. The large variety of types of bacteria found in breast tissue in former studies might also be a contamination of bacteria during surgical removal and laboratory investigation.

This is the first study in which breast implants have been studied using a highly sensitive PCR assay. Nonetheless, there are some limitations to the present study. One of the limitations is the heterogeneity of the research population; the primary operation indication in most females was cosmetic, but we also included two females who primarily underwent breast reconstruction. Furthermore, this was a cross-sectional study instead of a prospective study, which resulted in a wide range of time to follow-up. The implant duration in this study is quite long, which means that it is difficult to determine whether bacteria were the trigger of capsular contraction in the early stage. Moreover, it is also possible that a period of bacteraemia is a trigger for contraction of the capsule. This transient period is, however, not measurable with our study design. Finally, although the capsule is the most likely site of a potential low-grade bacterial infection, it is also possible that bacteria inhibit other breast tissue or implant sites. Additionally, we analysed only one 4-mm sample of the capsule, as described by the IS-pro technique, which may not be representative of the entire capsule.

It should be noted that capsular contracture is likely a multifactorial condition. In addition to the role of bacterial contamination, it is suggested that an alteration in the immune system might cause capsular contracture [2, 49–59]. Several studies have investigated the role of the immune system [2, 49–59]. Nonetheless, these studies involved immune factors that are upregulated in different stages in different pathways of the immune cascade. The exact immune cascade leading to capsular contracture is therefore still unknown. To investigate this immune cascade, it would be essential to investigate host–graft interactions, such as toll-like receptor upregulation [60]. Many studies also suggest a role for several patient-, surgery- and implant-related risk factors [5, 7, 12, 61–66]. For example, a longer duration of implantation, breast reconstructive surgery in patients with a history of breast cancer, subglandular implant placement, postoperative haematoma and a textured implant surface have shown a presumptive increased risk in the development of capsular contracture [15]. Little, weak or no evidence was found for the following factors: incision location; mean age at surgery; BMI; smoking; infection; preoperative antibiotic irrigation; complications such as granuloma, seroma or abscess; implant manufacturer, volume, shape, and content; implant rupture; and gel leakage. Study populations investigating these factors were, however, too heterogeneous, containing too many different variables in divergent proportions. Moreover, the Baker classification is inconsistently defined between studies. A clear, worldwide, consistently used classification for capsular contracture is needed to study the prevalence of capsular contracture in the future.

In order to obtain a better understanding of the aetiopathogenesis of capsular contracture, it is important to investigate the role of the abovementioned factors in a prospective multivariate study consisting of immunobiological as well as patient-, surgery- and implant-related risk factors.

## Conclusion

The present study shows, using a highly sensitive PCR assay, that breast capsules and glandular breast tissue are generally sterile. Moreover, no relationship was found between the presence of bacteria and the Baker score. Since all of the bacteria that were found were commensal skin bacteria, we believe that their presence might originate from contamination of the capsules during removal. However, in addition to the current PCR assay, more studies including larger sample sizes and samples taken from more sites of the capsule need to be conducted to further investigate the presence of bacteria in capsular contracture.

Further research is needed to investigate the aetiopathogenesis of capsular contracture. Special attention must be paid to any possible alteration in the immune system. Additionally, patient, implant and surgical factors must be investigated in a prospective multivariate study.
